# Cerebrospinal Fluid Metabolomics Identified Ongoing Analgesic Medication in Neuropathic Pain Patients

**DOI:** 10.3390/biomedicines11092525

**Published:** 2023-09-13

**Authors:** Emmanuel Bäckryd, Katarina Thordeman, Björn Gerdle, Bijar Ghafouri

**Affiliations:** Pain and Rehabilitation Center, and Department of Health, Medicine and Caring Sciences, Linköping University, Linköping, Sweden

**Keywords:** acetaminophen, analgesics, biomarkers, chronic, CSF, neuropathic, metabolomics, pain

## Abstract

Background: Cerebrospinal fluid (CSF) can reasonably be hypothesized to mirror central nervous system pathophysiology in chronic pain conditions. Metabolites are small organic molecules with a low molecular weight. They are the downstream products of genes, transcripts and enzyme functions, and their levels can mirror diseased metabolic pathways. The aim of this metabolomic study was to compare the CSF of patients with chronic neuropathic pain (*n* = 16) to healthy controls (*n* = 12). Methods: Nuclear magnetic resonance spectroscopy was used for analysis of the CSF metabolome. Multivariate data analysis by projection discriminant analysis (OPLS-DA) was used to separate information from noise and minimize the multiple testing problem. Results: The significant OPLS-DA model identified 26 features out of 215 as important for group separation (R^2^ = 0.70, Q^2^ = 0.42, *p* = 0.017 by CV-ANOVA; 2 components). Twenty-one out of twenty-six features were statistically significant when comparing the two groups by univariate statistics and remained significant at a false discovery rate of 10%. For six out of the top ten metabolite features, the features were absent in all healthy controls. However, these features were related to medication, mainly acetaminophen (=paracetamol), and not to pathophysiological processes. Conclusion: CSF metabolomics was a sensitive method to detect ongoing analgesic medication, especially acetaminophen.

## 1. Introduction

To better understand the pathophysiology of chronic pain and bridge the translational gap between animals and humans [1,2,3], cerebrospinal fluid (CSF) is a sensible biofluid to investigate. CSF can reasonably be hypothesized to mirror central nervous system (CNS) pathology. We have previously found evidence of neuroinflammation when analyzing CSF from fibromyalgia patients [4] and neuropathic pain patients [5], compared to healthy controls. This is in line with growing evidence of an interplay between the immune and nervous systems [2,6,7]. In a CSF proteomic study, we also found that an isoform of angiotensinogen had the highest power to separate neuropathic pain patients from healthy controls in a multivariate model [8]. This finding confirmed results from animal models and clinical trials concerning the possible involvement of the renin–angiotensin system in neuropathic pain [9,10]. Hence, CSF biomarker studies seem promising for animal-to-human translation and backtranslation. However, the difficulty in sampling CSF is an obvious drawback, especially concerning healthy controls. 

Neuropathic pain is caused by a lesion or disease of the somatosensory nervous system [11]. Advances in basic science using animal models have not translated into better treatments for neuropathic pain [3]. Available analgesics often have limited effects or lead to troublesome side-effects [12,13]. Current evidence indicates that at least six patients have to be treated with a first-line drug in order for one patient to obtain clinically significant pain relief [13], i.e., numbers needed to treat (NNT) ≥ 6. 

Untargeted omics methods, such as proteomics [14] or metabolomics [15], can be used to explore the pathophysiological mechanisms of neuropathic pain. Metabolomics deals with the identification and quantification of small molecules—metabolites. Metabolites are small organic molecules with a low molecular weight. They are the downstream products of genes, transcripts and enzyme functions, and hence their levels may mirror normal or diseased metabolic pathways [15]. Knowledge about metabolic pathways activated in different chronic pain conditions can be helpful for the development of new analgesics. 

Nuclear magnetic resonance (NMR) spectroscopy is a useful technique for the metabolomic study of different diseases including chronic pain conditions, such as neuropathic pain or fibromyalgia. NMR enables the detection of several hundred small molecules in body fluids such as CSF, blood or urine [15,16,17,18,19]. In a previous NMR metabolomic study, we compared blood from patients suffering from chronic neuropathic pain with healthy controls, finding that several of the metabolites that significantly differed between groups were involved in inflammatory processes, while others were important for CNS functioning and neural signaling [16]. 

The aim of this explorative, observational, cross-sectional study was to investigate the CNS pathophysiology of neuropathic pain by comparing the CSF metabolome of patients with healthy controls, using multivariate data analysis by projection (MVDA) to analyze the correlation structure of the material, thereby separating information from noise and minimizing the multiple testing problem [20,21]. We also wanted to investigate if pain intensity and/or pain duration were associated with metabolomic patterns. 

## 2. Materials and Methods

### 2.1. Participants 

Twelve healthy individuals were recruited as healthy controls, as described previously [8]. Chronic neuropathic pain patients (*n* = 16) were included in the study. All patients were recruited by convenience sampling from an open-label clinical trial evaluating the effect of bolus injections of ziconotide [22]. Immediately before ziconotide injection, blood and CSF samples were collected. Patients had to be refractory to conventional anti-neuropathic pain pharmacological treatment and were under consideration for spinal cord stimulation (SCS) at Linköping University Hospital, Sweden. 

Inclusion criteria were as follows: patient at least 18 years of age; chronic peripheral neuropathic pain (6 months or more) caused by trauma or surgery for whom conventional pharmacological treatment had failed; average visual analogue scale (VAS) pain intensity last week of 40 mm or more; patient capable of judgement, meaning the patient was able to comprehend information about the drug, its administration and evaluation of efficacy and side effects; signed informed consent. 

Exclusion criteria were: intrathecal chemotherapy; pregnant or lactating women; limited life expectancy; intracranial hypertension; known liver or kidney disease characterized by serum transaminases, total bilirubin, alkaline phosphatase or creatinine > 1.2 times above the upper normal limit; advanced cardiopulmonary disease, ongoing infection in the lumbar area (systematically or locally), coagulopathy; history of psychiatric disorder; allergy to ziconotide or any of the excipients in the ziconotide vial; participation in another clinical trial during the last 30 days. 

As reported in previously published papers [8,16,22,23], the nerve injury that was deemed to cause the neuropathic pain was classified using the International Classification of Diseases version 10 (ICD-10) (see Table 1). Four patients suffered from peripheral neuropathic pain projected to the upper extremity, and twelve from peripheral neuropathic pain projected to the lower extremity. All patients included in this study described their pain as continuous. Twelve of the patients experienced an exacerbation of the pain during physical activity. Nine of the patients had concomitant diseases: hypertension (*n* = 4), polymyalgia rheumatica (*n* = 1), psoriasis (*n* = 2), fibromyalgia (*n* = 1), diabetes (*n* = 1), mild angina (*n* = 2), panic disorder (*n* = 1), mild obstructive lung disease (*n* = 1), vertebral compressions (*n* = 1), orthostatism (*n* = 1), peptic ulcer (*n* = 1), dyspepsia (*n* = 1) and anemia (*n* = 1). The use of concomitant medication including analgesics was also reported. Four of the patients no longer used any pain medication at inclusion, while three patients had a single analgesic and nine patients used two analgesics or more. The distribution of the usage of the different medications were paracetamol (*n* = 7), non-steroidal anti-inflammatory drugs (NSAID) (*n* = 2), opioids (*n* = 9), gabapentinoids (*n* = 7) and antidepressants (*n* = 6). 

Participants answered questionnaires about pain and their state of health. We registered basic background information such as sex, age, weight, length, pain duration (measured in months), average pain intensity (VAS), location of pain, characterization of pain (constant, intermittent or transitory), impact of physical activity on pain, concomitant diseases and ongoing medication.

### 2.2. Sample Collection

A lumbar puncture was performed by an experienced anesthesiologist (EB) with a 27 GA pencil-point Whitacre needle (BD, Franklin Lakes, NJ, USA), and a 10 mL sample of CSF was drawn in five numbered syringes of 2 mL each. Each sample was immediately cooled on ice, transported to the Painomics^®^ laboratory, Linköping University Hospital, centrifuged, divided in aliquots and stored at −70 °C. 

### 2.3. Metabolomics Analysis

CSF samples was mixed (5:1) with phosphate buffer solution containing 1.5 M KH_2_PO_4_, 580 µM TSP-d4, NaN_3_, D_2_O, pH 7.4 and transferred to 3 mm NMR tubes. The samples were analyzed at the Swedish Nuclear Magnetic Resonance (NMR) Centre in Gothenburg using an Oxford 800 Mhz magnet equipped with a Bruker Avance III HD console, 3 mm TCI cryoprobe and a cooled Sample Jet auto sampler (Bruker BioSpin, Fällanden, Switzerland), as described previously [16]. Generated data from NMR spectrometer were processed in TopSpin3.5pl7 (Bruker BioSpin, Fällanden, Switzerland). Chenomx 9.0 (Chenomx Inc., Edmonton, AB, Canada) and the Human Metabolite Database [24] were used for identification of the metabolite signals.

### 2.4. Statistics

IBM SPSS Statistics (version 27.0, IBM, Armonk, NY, USA) was used for bivariate analyses. Unless stated otherwise, data are presented as median (25th–75th percentile). Mann–Whitney U test and Chi square test were used as appropriate for group comparisons. Spearman’s rho was calculated for bivariate correlations. A *p*-value ≤ 0.05 was considered significant. To handle the multiple testing problem, a false discovery rate (FDR) at the 10% level was applied using the Benjamini–Hochberg procedure [25]. 

Multivariate data analysis (MVDA) by projection was performed on metabolomics data with SIMCA-P+ (v.17.0, Sartorius Stedim Biotech, Umeå, Sweden). First, principal component analysis (PCA) was used for quality control and to check for multivariate outliers (strong outliers defined as Hotelling’s T2 >> T2Crit (95%) and moderate outliers as DModX > 2 *DCrit). Then, we effectuated orthogonal partial least squares—discriminant analysis (OPLS-DA)—whereby group belonging was used as the outcome variable (Y-variable) and the metabolic features as predictors (X-variables). OPLS was used instead of OPLS-DA when non-categorical variables were used as Y-variables (such as VAS pain intensity, pain duration, age or body mass index (BMI)). The parameters used for evaluation of the OPLS-DA and OPLS models were R^2^ (goodness of fit), Q^2^ (goodness of prediction) and cross validated analysis of variance (CV-ANOVA) which provides a familiar metric for the model as a whole (i.e., a *p*-value). The differences between R^2^ and Q^2^ should not be greater than 0.3, otherwise suggesting overfitting. The variable influence on projection (VIP) indicates the relevance of the group of X-variables that best explain Y. A backward variable elimination strategy was applied [26], whereby the 20 X-variables with the lowest VIP were eliminated and a new OPLS-DA model was built. This procedure was reiterated until Q^2^ stopped increasing, indicating an optimal model. We also used p(corr) which is the loading of each variable scaled as a correlation coefficient (range from −1 to +1). VIP ≥ 1.0, absolute p(corr) ≥ 0.6 and CV-ANOVA ≤ 0.05 were considered as significant. The analysis and the presented parameters are in accordance with the guidelines presented by Wheelock and Wheelock [20]. 

### 2.5. Ethics

The regional ethics committee in Linköping approved the study (Dnr M136-06 and Dnr 2012/94-32). The study was conducted in accordance with the Helsinki Declaration. After verbal and written information, written informed consent was obtained from all the participants. 

## 3. Results

### 3.1. Background Data

The patient group consisted of 36% females and the healthy control group of 58% females; the difference between groups was not statistically significant (*p* = 0.249). Patients were older than controls (57.5 (52.0–69.0) vs. 51.0 (27.5–54.5) years, *p* = 0.005). Patients also had a tendency towards higher BMI (26.5 (23.3–29.7) vs. 23.9 (22.0–25.4) kg/m^2^, *p* = 0.072). VAS pain intensity in patients was 72 (59–82) mm and pain duration 59 (36–120) months. 

### 3.2. Overview and Quality Control of Metabolic Data 

First, an unsupervised PCA model was computed for overview and quality control of the data using 444 metabolomic features as X-variables (*n* = 28, 444 X-variables, R^2^ = 0.49, Q^2^ = 0.27). Two patients were identified as strong outliers and were therefore excluded from further analyses. As shown in the Supplementary Materials S1, the contribution plot of one of them revealed that the features contributing most to the position of that individual in the PCA model were almost all related to glucose—and this was indeed the patient with concomitant diabetes (see Section 2.1).

### 3.3. OPLS-DA Model Comparing Patients and Controls

**Figure 1:**
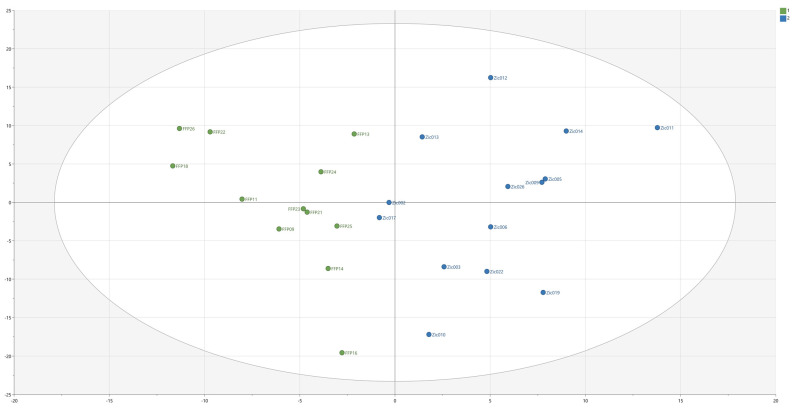
Score plot of OPLS-DA model, showing group separation between patients and controls along the first predictive component (x-axis). Each patient is a blue dot, each healthy control is a green.

After an initial OPLS-DA model with *n* = 26, 2 components (the first predictive and the second orthogonal), 444 X-variables, R^2^ = 0.74, Q^2^ = 0.30 and *p* = 0.101 by CV-ANOVA, a backward variable elimination strategy was applied, resulting in a final OPLS-DA model with *n* = 26, 2 components (the first predictive and the second orthogonal), 215 X-variables, R^2^ = 0.70, Q^2^ = 0.42, *p* = 0.017 by CV-ANOVA. The score plot of the model is depicted in Figure 1, illustrating clear group separation. The model was additionally validated using a permutation plot (Supplementary Materials S2). In total, 26 features had an absolute p(corr) ≥ 0.6 for the first predictive component, indicating that they, when taking the whole correlation structure of the material into consideration, were the most important variables for group separation (Table 2). Twenty-one out of twenty-six features were statistically significant when comparing the two groups by univariate statistics (Table 2), and all twenty-one remained significant when a FDR of 10% was applied. Notably, for six out of the top ten metabolomic features listed Table 2, the features were absent in *all* healthy controls. The six features only identified in patients were 26_C, 41_C, 40_C, 29_C, 30_C and 36_C. 

The top 10 features of Table 2 were then analyzed in more depth, focusing on patients (*n* = 14). A bivariate correlation matrix for the top 10 features is presented in Table 3, showing that 8 of the features demonstrated a high degree of intercorrelation (the exceptions being 450_C and 503_C). This pattern was confirmed by the loading plot of the OPLS-DA model, part of which is depicted in Supplementary Materials S3. Going back to Table 3 and looking specifically at the six features absent in healthy controls, all bivariate correlation coefficients except for 36_C ranged 0.789–0.995, i.e., there was (in patients) a very high degree of intercorrelation between five features that were absent in healthy controls. 

Then, the features tabulated in Table 2 were tentatively annotated, revealing that most of them were related to medication, mainly acetaminophen (paracetamol); see Table 2. When the features related to medication were removed from the above-mentioned OPLS-DA model, it was no longer significant (*n* = 26, 1 component, 197 X-variables, R^2^ = 0.29, Q^2^ = 0.18, *p* = 0.10 by CV-ANOVA).

### 3.4. OPLS Models in Patients

In patients (*n* = 14), it was not possible to regress VAS by OPLS using the 444 features as in the OPLS-DA model above as X-variables. Likewise, a non-significant model resulted when regressing pain duration by OPLS (*n* = 14, 2 components, R^2^ = 0.85 and Q^2^ = 0.21, *p* = 0.68 by CV-ANOVA) with the same 444 features. 

Additionally, to investigate a potential confounding effect of age (which different significantly between groups) on the main OPLS-DA results as per the above, an OPLS model with age as outcome variable (Y-variable) was computed using the 215 features of the final OPLS-DA model as X-variables. The initial model had *n* = 14, R^2^ = 0.87, Q^2^ = 0.40 and *p* = 0.28 by CV-ANOVA. After the backward elimination procedure, the final model had 174 X-variables, *n* = 14, R^2^ = 0.82, Q^2^ = 0.44 and *p* = 0.21 by CV-ANOVA. Notwithstanding the fact that the OPLS model was not significant, we still scrutinized the absolute p(corr) values for the 12 features listed in Table 2 that remained in the OPLS model after the backward elimination procedure; none of them showed any indication of being correlated to age (all had absolute p(corr) ≤ 0.45). Moreover, using bivariate statistics, none of the features listed in Table 2 had any significant correlation to age. Hence, we found no indication that our findings in Table 2 were primarily related to age rather than to belonging to a specific group. 

Finally, in a manner similar to age, we also investigated a potential confounding effect of sex on the main OPLS-DA results. After the backward elimination procedure, the final model with sex as Y variable had 175 X-variables, *n* = 14, R^2^ = 0.37, Q^2^ = 0.20 and *p* = 0.29 by CV-ANOVA. Notwithstanding the fact that the model was statistically non-significant, we still scrutinized the absolute p(corr) values for the eight features listed in Table 2 that remained in the model after the backward elimination procedure; only one of them, namely ala + fructose_205_C, showed an indication of being correlated to sex (absolute p(corr) = 0.78). We therefore compared ala + fructose_205_C in men vs. women, finding a statistically non-significant tendency towards higher levels of ala + fructose_205_C in men (*p* = 0.125, see Supplementary Materials S4). 

## 4. Discussion

Unexpectedly, the main finding of the study was that CSF metabolomics was a sensitive method to detect ongoing analgesic medication, especially acetaminophen (also known as paracetamol), in chronic pain patients. We wish to highlight three aspects of this “negative” result. 

First, our findings are a reminder of the importance of collecting information about concomitant medication when conducting pain biomarker studies in general, and perhaps in particular when studying CSF. In correlative studies such as this one, several confounding factors may be at work simultaneously. Ideally, controls should be age-, BMI- and sex-matched. However, this is especially difficult in CSF studies, given the difficulty in obtaining CSF in contrast to blood or saliva. Other possible confounders could be concomitant diseases or concomitant medication—and the latter seems to be the case in the present study. 

Second, although in a sense disappointing, our results clearly demonstrate the power of CSF metabolomics combined with MVDA in identifying real and relevant group differences for central nervous system processes. Given that modern omics studies can generate a vast amount of data (meaning that the number of variables can by far exceed the number of participants), the omics field actualizes the multiple testing problem—hence, the potential accusation of being on a fishing expedition, also known as data dredging or p-hacking. Indeed, the phenomenon of alpha inflation means that if two groups are compared two times, the risk of getting at least one significant result due to chance is not 5%, but almost 10%. If instead 20 comparisons are made, the probability increases to 64%; this phenomenon is captured by the formula 1 − 0.95^k^, where k is the number of comparisons [27,28]. If 100 comparisons are made, the probability hence rises to 99%. While we do understand and concur with such concerns, we also think that explorative studies such as this one are important to conduct, and that this can be sensibly achieved. One crucial aspect is the possibility of using not only traditional univariate statistics but MVDA, thereby taking the whole correlation structure of the material into consideration, hence better separating the valuable information from the noise [21]. MVDA is of course no panacea, but it is an important tool [20]. If we go back to the present study, it is notable that the combination of omics (in this case, metabolomics) and MVDA was able to detect a difference between the groups that we indeed know to be true (i.e., patients were on medication). We think that this should give researchers in the field some level of confidence that similar proteomics or proteomics-related studies using this statistical methodology can generate true results.

Third, our results are in line with the view that acetaminophen acts in the CNS. Until quite recently, at least in Sweden, the standard textbook teaching was that acetaminophen is a *peripherally* acting analgesic [29]. After more than 100 years of use, the exact mechanism of action of paracetamol remains to be determined [30]. In the CNS, acetaminophen has been said to have effects on cyclooxygenase, on serotonergic descending neuronal pathways, on L-arginine/NO pathways and on the endocannabinoid system [31]. Alternatively, acetaminophen analgesia could be mediated by the formation of its bioactive AM404 metabolite in the central nervous system [32]. Even if our findings do not prove that acetaminophen is centrally acting (it could be an epiphenomenon), they are nonetheless highly congruent with that view. 

There are few NMR metabolomics studies on neuropathic pain in general, and on CSF in particular. We have previously reported interesting NMR metabolomic findings in blood from the same patients with chronic neuropathic pain [16]. In that study, 50 out of 326 features in blood significantly contributed to group separation between patients and healthy controls, the significant metabolites being involved in inflammation, CNS functioning and neural signaling [16]. In the present CSF study, the “metabolomic trace” of the treatment given to patients had a much higher magnitude than differences due to chronic pain pathophysiology. Therefore, should patients taking analgesics be excluded in future CSF metabolomic studies? The difficulty in obtaining CSF would be an argument in that direction, i.e., given the low number of patients included in CSF studies, group homogeneity is all the more important when studying CSF as opposed to blood or saliva (which are more easily collected). 

## 5. Limitations

The low number of participants in this observational study is an obvious limitation. However, it is important to understand that studies such as the present one are not intended to generate *clinical* biomarker candidates. If that had been our purpose, dozens or perhaps hundreds of samples would have been necessary. Instead, using the terminology proposed by Pavlou et al., this was an early discovery phase, pre-clinical exploratory study [33]. For such studies, in which the aim is to strive towards a better understanding of pathophysiological mechanisms in humans, the study design requirements are different from clinical biomarker studies [34]. Another limitation pertains to the fact that, as already mentioned, it would have been better to have age-matched controls. Also, given the small sample size, a narrower age range would have been preferable (the age range of all participants was 21–75 years). 

## 6. Conclusions

We have shown that the combination of CSF metabolomics and MVDA is a powerful tool to detect ongoing molecular events in the central nervous system. We were unable to detect a disease signal in patients with chronic neuropathic pain vs. healthy controls, centrally acting medication overshadowing the putative pathophysiologically interesting findings. These negative results notwithstanding, CSF metabolomics still have a role to play when investigating the mechanisms of chronic pain; however, our study shows that the power of an investigative method can also be its problem. 

## Figures and Tables

**Table 1:** Nerve injury according to the ICD-10 classification system (*n* = 16) in falling order of frequency.

ICD-10 Code	Nerve Structure	Number of Participants
S342	Root of lumbar or sacral spine	8
S142	Root of cervical spine	3
S740	Sciatic nerve at hip and thigh level	1
S549	Unspecified nerve at forearm level	1
S949	Unspecified nerve at ankle or foot level	1
S841	Peroneal nerve at lower leg level	1
S343	ICauda equina	1
G629	Polyneuropathy, unspecified	1

**Table 2:** Cerebrospinal metabolomic features separating chronic neuropathic pain patients (*n* = 14) from healthy controls (*n* = 12).

Feature ID	Tentative Annotation	Pain Patients Intensity	Healthy Controls Intensity	p(corr)	VIP	*p*-Value
_43_C	Tyrosine + acetaminophen	46,954 (40,742–58,404)	36,427 (34,508–40,219)	0.81	1.48	0.041 *
_26_C	Acetaminophen	35,906 (0–59,894)	0 (0–0)	0.79	1.48	0.001 *
_44_C	Acetaminophen + tyrosine	54,407 (17,385–86,355)	15,232 (11,381–16,918)	0.79	1.47	0.005 *
_41_C	Acetaminophen	14,400 (0–26,768)	0 (0–0)	0.76	1.42	0.005 *
_40_C	Acetaminophen	42,075 (0–66,636)	0 (0–0)	0.76	1.42	0.005 *
_29_C	Acetaminophen	34,118 (0–59,041)	0 (0–0)	0.76	1.42	0.005 *
_30_C	Acetaminophen	5111 (0–14,300)	0 (0–0)	0.73	1.35	0.010 *
_450_C	Unknown singlet; acetamide?	58,600 (43,259–65,879)	39,481 (34,774–43,149)	0.73	1.34	0.005 *
_36_C	Unknown; diclofenac?	10,885 (0–16,224)	0 (0–0)	0.73	1.33	0.005 *
_503_C	Unknown singlet	37,373 (23,829–65,360)	25,998 (18,988–29,115)	0.72	1.32	0.020 *
_416_C	Acetaminophen	122,276 (98,341–146,314)	91,822 (84,232–97,395)	0.71	1.29	0.023 *
_333_C		23558 (21,121–26,910)	20,066 (14,804–23,496)	0.70	1.30	0.150
_21_C	Unknown; diclofenac?	5046 (0–12,759)	0 (0–0)	0.69	1.25	0.010 *
_352_C	Unknown singlet; dimethylamine?	66,322 (56,259–74,161)	51,341 (45,414–62,591)	0.68	1.25	0.051
_546_C	Pregabalin?	23,466 (20,505–49,519)	17,954 (15,269–19,301)	0.67	1.21	0.003 *
_396_C	Unknown singlet; acetylsalicylate?	17,326 (14,477–23,720)	13,263 (12,062–14,112)	0.67	1.21	0.007 *
_28_C	Acetaminophen	5756 (0–18,558)	0 (0–0)	0.67	1.24	0.010 *
_322_C		23,721 (21,822–25,907)	21,357 (13,720–22,045)	0.66	1.24	0.086
_502_C	Unknown singlet	19,648 (16,848–30,175)	15,496 (5388–17,337)	0.66	1.20	0.023 *
_543_C	Pregabalin?	20,593 (16,462–42,854)	15,809 (14,127–20,877)	0.65	1.17	0.014 *
_339_C	Unknown singlet; N,N-dimethylglycine?	12,799 (10,537–15,528)	0 (0–5595)	0.64	1.16	0.007 *
_35_C	Unknown; diclofenac?	5230 (0–12,274)	0 (0–0)	0.63	1.18	0.010 *
tyr_33_C	Tyrosine	35,814 (30,869–39,163)	32,743 (28,207–33,807)	0.62	1.13	0.114
_545_C	Pregabalin?	20,732 (14,992–50,151)	16,375 (15,239–18,050)	0.62	1.12	0.041 *
_548_C	Pregabalin?	20,748 (18,226–47,331)	18,624 (15,957–22,198)	0.61	1.10	0.063
ala + fructose_205_C	ALANINE + fructose	248,350 (214,837–268,264)	199,950 (190,866–223,795)	0.60	1.22	0.020 *

The metabolites are listed in descending order of absolute p(corr). A positive p(corr) indicates higher levels in patients than in healthy controls, while a negative p(corr) indicates the opposite. Intensity is expressed as median (IQR). * denotes significance at the 0.05 level; all remained significant at a false discovery rate (FDR) of 10%. The suffix C denotes cerebrospinal fluid.

**Table 3:** Correlation matrix in *patients* (*n* = 14) by Spearman’s rho for the top 10 features separating patients from healthy controls.

		_43_C	_26_C	_44_C	_41_C	_40_C	_29_C	_30_C	_450_C	_36_C	_503_C
_43_C	Rho	1.000	0.856 **	0.842 **	0.867 **	0.881 **	0.872 **	0.777 **	0.284	0.540 *	0.424
	*p*-value		<0.001	<0.001	<0.001	<0.001	<0.001	0.001	0.326	0.046	0.131
_26_C	Rho		1.000	0.960 **	0.978 **	0.973 **	0.983 **	0.823 **	0.290	0.571 *	0.254
	*p*-value			<0.001	<0.001	<0.001	<0.001	<0.001	0.315	0.033	0.381
_44_C	Rho			1.000	0.947 **	0.956 **	0.956 **	0.819 **	0.270	0.641 *	0.160
	*p*-value				<0.001	<0.001	<0.001	<0.001	0.350	0.014	0.584
_41_C	Rho				1.000	0.981 **	0.995 **	0.779 **	0.204	0.581 *	0.190
	*p*-value					<0.001	<0.001	0.001	0.485	0.029	0.516
_40_C	Rho					1.000	0.990 **	0.828 **	0.213	0.562 *	0.199
	*p*-value						<0.001	<0.001	0.465	0.037	0.495
_29_C	Rho						1.000	0.789 **	0.194	0.557 *	0.199
	*p*-value							0.001	0.505	0.038	0.495
_30_C	Rho							1.000	0.406	0.574 *	0.289
	*p*-value								0.150	0.032	0.317
_450_C	Rho								1.000	0.384	0.486
	*p*-value									0.175	0.078
_36_C	Rho									1.000	0.318
	*p*-value										0.268
_503_C	Rho										1.000
	*p*-value										

Correlation coefficients in bold. * denotes significance at the 0.05 level and ** denotes significance at the 0.001 level. The feature id suffix C denotes cerebrospinal fluid.

## Data Availability

Data are not publicly available.

## Supplementary Materials

The following supporting information can be downloaded at: https://www.mdpi.com/article/10.3390/biomedicines11092525/s1.
