# Supplementary material for: Cerebrospinal Fluid Metabolomics Identified Ongoing Analgesic Medication in Neuropathic Pain Patients
Source: Biomedicines. 2023 Sep 13;11(9):2525. doi: 10.3390/biomedicines11092525 (PMC10526053; doi:10.3390/biomedicines11092525)
Supplement: Supplementary file 1 [file biomedicines-11-02525-s001.zip › biomedicines-2500033-supplementary.pdf]

Supplementary Materials S1

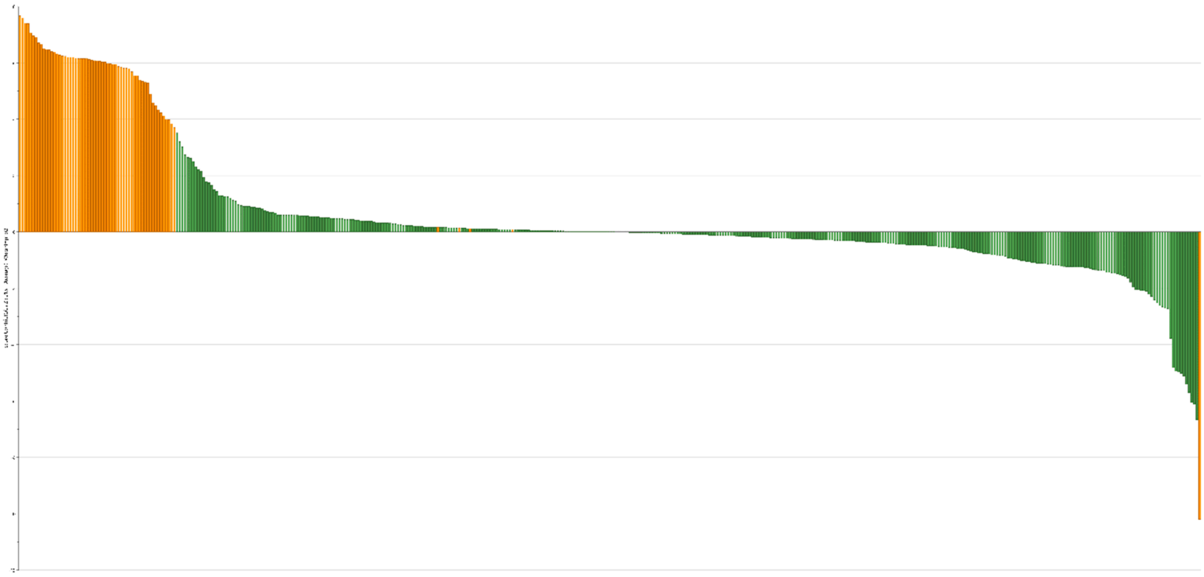

Above is a **contribution plot for patient Zic015**. Each column represents a X-variable, i.e., each column expresses the score contribution (called Weight) of each variable expressed as the difference between the individual in question and the average. Coloring is by SIMCA-default, orange columns showing the variables that contribute the most for Zic015. Below, for purposes of interpretation, we list the most important ("orange") variables, in falling order of Weight. Note that glc=glucose and that the suffix C denotes cerebrospinal fluid:

Name of X-variable	Weight
beta-glc (attenuated)_67_C	7.65321
glc+gln_211_C	7.58669
_493_C	7.03222
beta-glc (attenuated)_68_C	6.90862
glc_285_C	6.73999
_491_C	6.6743
_492_C	6.66529
glc_217_C	6.63557
glc_276_C	6.55021
alpha-glc_56_C	6.43098
glc+fructose_173_C	6.42943
alpha-glc_57_C	6.33893
glc_277_C	6.31861
_298_C	6.27931
glc+fructose_176_C	6.20533
glc_275_C	6.20264
glc_274_C	6.19837
glc_194_C	6.17351
glc+fructose_174_C	6.16794
glc_219_C	6.15058

glc+fructose_177_C	6.1368
glc_278_C	6.09901
glc_271_C	6.0944
glc_196_C	6.08112
glc_189_C	6.06117
glc_218_C	6.04696
glc_190_C	6.03791
glc_309_C	6.00169
glc_199_C	5.98645
glc_200_C	5.98247
glc+fructose_222_C	5.94988
glc_216_C	5.94664
glc_307_C	5.92836
glc_215_C	5.90836
glc_269_C	5.87302
glc_310_C	5.85255
glc_272_C	5.85083
glc_273_C	5.85083

Hence, almost all important variable in the contribution plot are associated with glucose.

Supplementary Materials S2

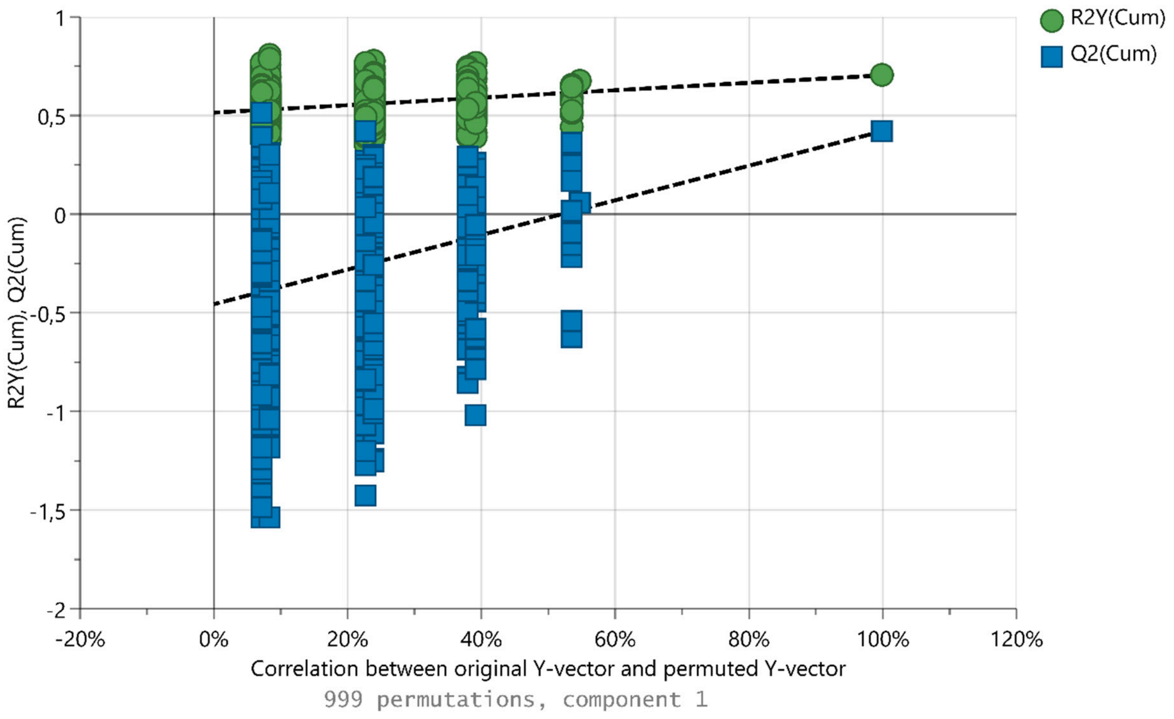

This is a permutation plot which confirms the validity of the OPLS-DA model, the intercept for Q2 being <0.

Supplementary Materials S3

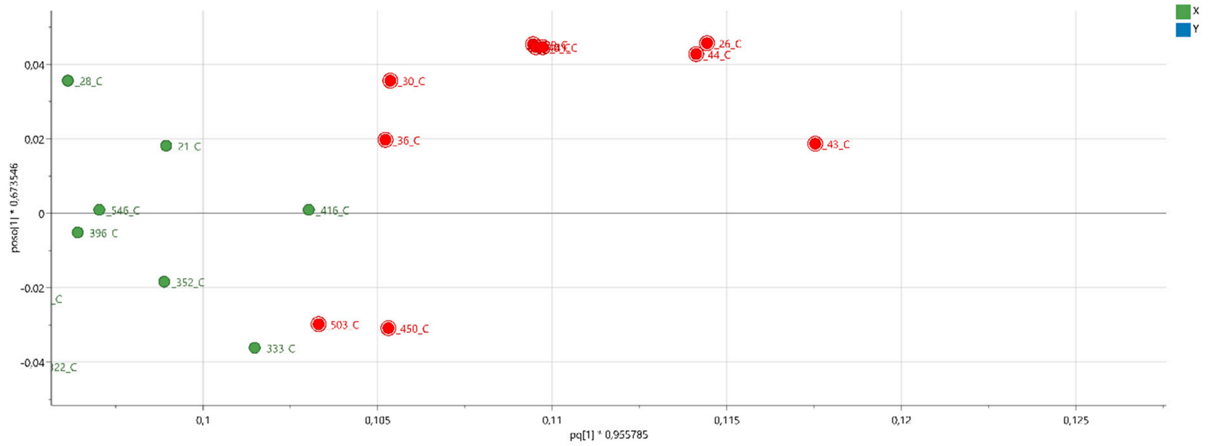

This is a zoomed-in part of the loading plot of the OPLS-DA model of patients vs. healthy controls. In other words, this is a part of the loading plot corresponding to the score plot depicted in **Figure 1** in the main text. The features mentioned in the paper are in red. Note especially the extremely high inter-correlation of 29_C, 40_C and 41_C; so much so that the three are almost merged in the plot. This is confirmed by the fact that the corresponding bivariate correlation coefficients for these three features range 0.981-0.995 (Spearman's rho, see **Table 3** in the main text).

Supplementary Materials S4

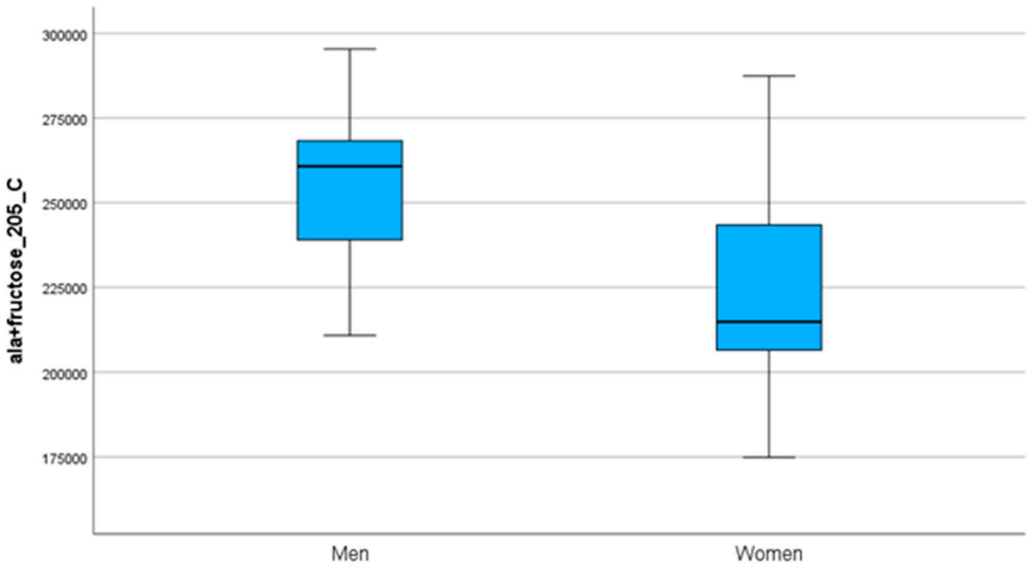

This box plot illustrates feature “ala+fructose_205_C” in patients, comparing men and women (p=0.125).
